# Long-Term Treatment with the Calcitonin Gene-Related Peptide Receptor Antagonist Erenumab in CADASIL: Two Case Reports

**DOI:** 10.3390/jcm13071870

**Published:** 2024-03-24

**Authors:** Maria Albanese, Francesca Pescini, Chiara Di Bonaventura, Luigi Francesco Iannone, Silvia Bianchi, Anna Poggesi, Mario Bengala, Nicola Biagio Mercuri, Francesco De Cesaris

**Affiliations:** 1 Headache Center, Neurology Unit, Tor Vergata University Hospital, 00133 Rome, Italy; maria.albanese@hotmail.it (M.A.); mercurin@med.uniroma2.it (N.B.M.); 2 Department of Systems Medicine, University of Rome Tor Vergata, 00133 Rome, Italy; 3 Stroke Unit, Emergency Department, AOU Careggi, 50134 Florence, Italy; anna.poggesi@unifi.it; 4 NEUROFARBA Department, University of Florence, 50121 Florence, Italy; 5 Department of Experimental and Clinical Medicine, University of Florence, 50121 Florence, Italy; chiara.dibonaventura@unifi.it; 6 Section of Clinical Pharmacology and Oncology, Department of Health Sciences, University of Florence, 50121 Florence, Italy; 7 Department of Medical Surgical and Neurological Sciences, University of Siena, 53100 Siena, Italy; bianchi22@unisi.it; 8 Tor Vergata University Hospital, Medical Genetics Unit, 00133 Rome, Italy; mario.bengala@ptvonline.it; 9 Headache Center and Clinical Pharmacology Unit, Careggi University Hospital, 50134 Florence, Italy; francesco.decesaris@aouc.unifi.it

**Keywords:** erenumab, CADASIL, migraine, *NOTCH3*, CGRP

## Abstract

Cerebral autosomal dominant arteriopathy with subcortical infarcts and leukoencephalopathy (CADASIL) is the most common monogenic form of cerebral small vessel disease, caused by a mutation in the *NOTCH3* gene on chromosome 19. The main clinical features include migraine (often with aura), early onset, recurrent subcortical ischemic strokes, mood disturbances, and cognitive impairment, frequently leading to dementia and disability with a reduction in life expectancy. Cerebral chronic global hypoperfusion, due to impaired cerebrovascular reactivity, seems to play a primary role in CADASIL. Migraine is the most common early feature of the disease, and to date, there are no consensus guidelines for treatment. Given the vasomodulatory influence of many antimigraine drugs, there is concern about their use in this disease. In particular, the calcitonin gene-related peptide (CGRP) system serves as a vasodilatory protective mechanism during cerebral and cardiac ischemia. Blocking this system could exacerbate ischemic events. Herein, we describe two CADASIL patients who were treated with the calcitonin gene-related peptide (CGRP) receptor antagonist erenumab for chronic migraine, reporting a significant reduction in the frequency of attacks and intensity of pain, and an improvement in quality of life without adverse effects.

## 1. Introduction

Cerebral autosomal dominant arteriopathy with subcortical infarcts and leukoencephalopathy (CADASIL; OMIM 125310) is the most common heritable cause of stroke and vascular dementia in adults and it is caused by *NOTCH3* gene mutations [1]. The *NOTCH3* gene encodes a transmembrane receptor protein mainly expressed on vascular smooth muscle cells (VSMCs), involved in vascular development and response to injury. *NOTCH3* mutations lead to structural microvascular abnormalities: wall thickening of small arteries and arterioles, disanchorage from the extracellular matrix, and progressive loss of VSMCs with subsequent breakdown of the integrity of the vascular wall [2], causing severe vasoreactivity impairment with subsequent reduced perfusion and tissue damage [3]. 

The earliest and most frequent findings on brain magnetic resonance imaging (MRI) are diffuse white matter hyperintensities (WMHs) in T2-weighted and fluid-attenuated inversion recovery (FLAIR) sequences; other typical alterations are multiple lacunes, dilated perivascular spaces, and microbleeds. Clinically, the disease is characterized by migraine, recurrent subcortical ischemic events, apathy, and other psychiatric disturbances, and progressive cognitive impairment mostly affecting executive functions [4]. Migraine is reported in about half of all CADASIL patients and is often the earliest feature of the disease [5], and constitutes the prominent symptom of CADASIL in some families [1]. 

The pattern of migraine in CADASIL differs from the general population, with a predominance of migraine with aura reaching a prevalence of 85%, which is nearly 10 times higher than that observed in the general population [6,7]. Usually, each patient presents more than one type of aura, and atypical symptoms are quite frequent (e.g., motor symptoms, basilar symptoms, confusion, alterations of consciousness or hallucinations) as well as prolonged episodes [1,5]. Moreover, acute reversible encephalopathy, often referred to as CADASIL coma, is another feature reported in about 10% of patients. The pathogenesis of aura in CADASIL is not yet known, but a reduction in the neuronal threshold to cortical spreading depression has been hypothesized [8]. In the CADASIL mouse model, this phenomenon is observed in the absence of a major reduction in cortical blood flow [4]. Contrary to previous beliefs, cerebral ischemia is probably not the primary mechanism of aura symptoms in CADASIL, while mutations in the *NOTCH3* gene appear to be responsible for early changes in cortical excitability in humans [9]. There is no consensus on acute and preventive treatments for migraine in these patients and limited data are available [10]. Since dysregulation of cerebrovascular reactivity seems to play an important role in the pathogenesis of CADASIL, there is concern regarding the use of common migraine preventive treatments mostly affecting cerebral vasoreactivity. In addition, migraine in CADASIL can be refractory to prophylactic therapies, thus making the therapeutic decision even more challenging [10]. Four anti-CGRP monoclonal antibodies (anti-CGRP mAbs) have been recently approved in Europe. One (erenumab) is directed against the CGRP receptor and three (galcanezumab, eptinezumab, and fremanezumab) against CGRP itself [11]. 

In the last year, several RCTs conducted with anti-CGRP mAbs have provided moderate to high-quality evidence recommending their use in individuals with episodic and chronic migraine. The initial guidelines advising the use of anti-CGRP mAbs categorized these treatments as third-line options, taking into consideration their cost [12]. However, in the last few updates, considering the increasing and robust data on their effectiveness and safety, anti-CGRP mAbs have been recommended as a first-line treatment [13,14]. All treatments for migraine and their place in therapy have been recently reviewed and discussed [15].

Herein, we report the outcomes of two CADASIL patients treated with erenumab for chronic migraine for a long period of time.

## 2. Patient 1 

**Figure 1:**
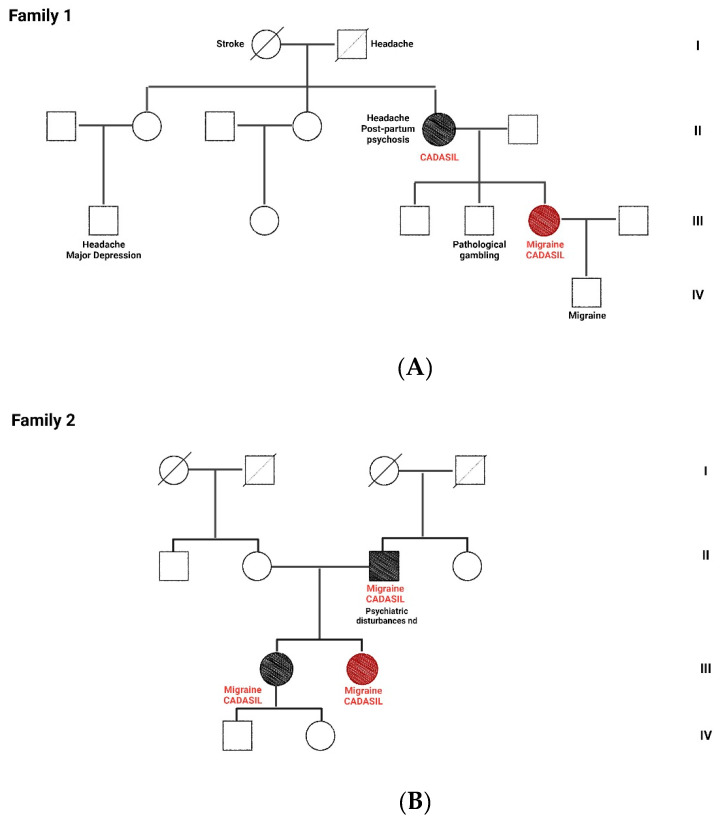
Family pedigrees of patients 1 (**A**) and 2 (**B**). I to IV are the reported generation for each family. Red-filled symbols indicate the probands, black-filled symbols represent family members with the genetic mutations in the *NOTCH3* gene. CADASIL symptoms are reported when present for each individual. Diagonal lines indicate deceased individuals. Circles are for female subjects and squares male subjects.

A 49-year-old female had a personal history of lactose intolerance, arterial hypertension controlled with an angiotensin receptor blocker, and no other relevant diseases. She had no cognitive impairment, neurologic deficits, or comorbid psychiatric diseases. Her family history showed a recurrence of headache and psychiatric disturbances (Figure 1A).

**Figure 2:**
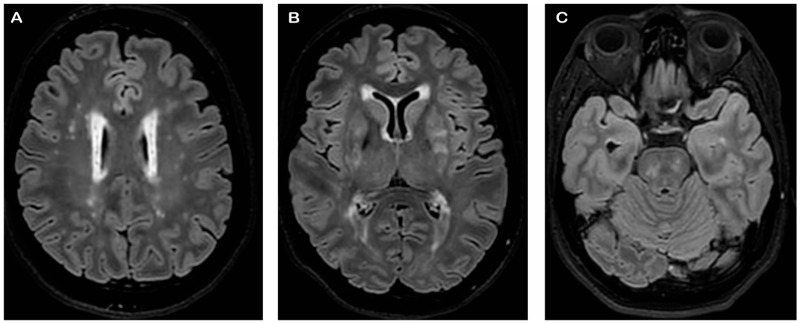
Patient 1’s FLAIR MRI axial plane showing multiple punctate hyperintensities in the deep white matter and confluent lesions in the periventricular white matter (**A**), minimal involvement of external capsules (**B**) and anterior temporal poles and more severe extension to the pons (**C**).

During the first visit in 2019 to the Headache Center, the patient reported headache onset at the age of eighteen and fulfilled the current diagnostic criteria for chronic migraine (15–17 attacks/month) without aura according to ICHD-3 [16]. Attacks consisted of moderate to severe bilateral pulsating pain, usually accompanied by phonophobia, photophobia, and the need to rest. Attacks were treated with triptans (i.e., rizatriptan 10 mg) or non-steroidal anti-inflammatory drugs (NSAIDs, i.e., ibuprofen 600 mg) often with full pain relief. The patient has been previously treated without clinical benefit with amitriptyline (up to 10 drops), topiramate (up to 100 mg daily), propranolol (40 mg daily), flunarizine (10 mg daily), and OnabotulinumtoxinA (up to 195 UI). During the following year, the patient was treated with venlafaxine (75 mg daily) and candesartan (8 mg daily) without advantage and a deterioration in migraine days (18–20 attacks/month). A very severe migraine-related disability was revealed using the Migraine Disability Assessment (MIDAS) questionnaire (score of 114) and the Headache Impact Test (HIT-6 score of 63). Treatment with erenumab 70 mg was started, and considering the drug-resistance of the migraine, a brain MRI was performed that showed multiple punctate WMHs in the deep white matter and confluent lesions in the periventricular white matter associated with enlarged perivascular spaces (Figure 2). At the three-month follow-up, the patient presented a drastic reduction in migraine days (12 days/months) and migraine-related disability (MIDAS score of 29 and HIT-6 score of 55). Due to the MRI findings, the patient was referred to an outpatient clinic dedicated to cerebral small vessel diseases. General and neurological examinations were normal. No coagulopathy or inflammatory disease was revealed by a blood and cerebrospinal fluid analysis. Analogously, no pathological findings were found on trans-thoracic echocardiography, carotid duplex ultrasound, visual evoked potentials, and cervical spine MR. The brain MRI was re-evaluated and minimal involvement of the anterior temporal poles and of the external capsules was noted. Considering the family and personal history and the MRI findings, CADASIL was suspected. Indeed, she scored 14 on the CADASIL scale [17] and a genetic test was conducted, confirming two pathogenic heterozygous *NOTCH3* gene mutations: *c.2042G>A (pCys681Tyr)* on exon 13 and *c3577T>C (pCys193Arg)* on exon 22. After 12 months of treatment, the patient reported to have migraine only eight days per month (thus reverted to episodic migraine) and a significant reduction in migraine disability burden with a MIDAS total score of 6 and a HIT-6 test score of 59. No adverse events have been described. The brain MRI was repeated, and no new lesions were found. Treatment with erenumab was continued due to the substantial clinical benefit and it is currently ongoing with persistent benefit.

## 3. Patient 2

**Figure 3:**
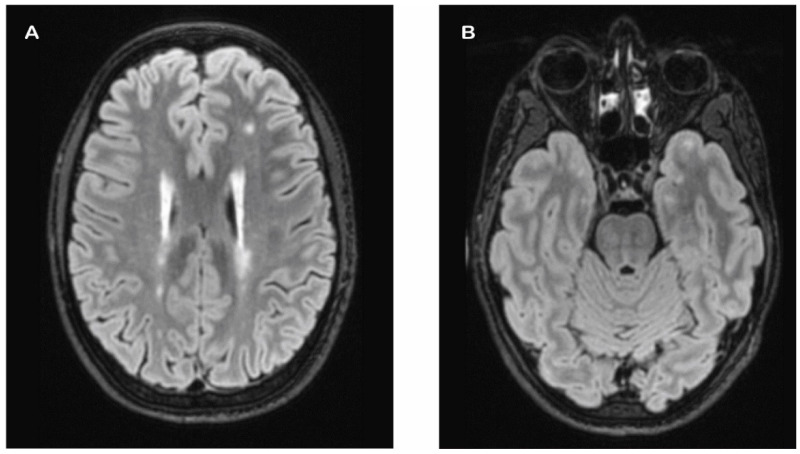
Patient 2’s FLAIR MRI axial plane showing multiple punctate hyperintensities in the deep white matter and confluent lesions in the periventricular white matter (**A**) and minimal involvement of anterior temporal poles (**B**).

A 31-year-old female of normal body weight (BMI 23 kg/m^2^) had a personal history of ovarian polycystosis, bruxism, and no other relevant diseases. She had no cognitive impairment, neurological deficits or comorbid psychiatric diseases. Her family history showed a recurrence of headache and cardiovascular disorders (Figure 1B). During her first visit in 2022 to our Headache Center, the patient reported recurrent headache since her childhood, and fulfilled the current diagnostic criteria for chronic migraine (15–18 attacks/month) with and without aura, according to ICHD-3 [16]. The attacks were characterized by bilateral pulsating pain of moderate–severe intensity, localized in the fronto-temporal region, often associated with photo- and phonophobia, nausea, and vomiting, lasting up to 72 h and frequently triggered by psychological stress and physical activity. The patient also reported episodes of aura twice a month, presenting as reversible sensorial and/or motor unilateral neurological symptoms. For acute therapy, the patient used oral triptans (i.e., almotriptan 12.5 mg) or non-steroidal anti-inflammatory drugs (ibuprofen 600 mg), with moderate success but with constant concomitant medication overuse. Different pharmacological and non-pharmacological preventive strategies were previously attempted (i.e., anticonvulsants, antidepressants, beta-blockers, onabotulinumtoxinA, ketogenic diet) and stopped due to a lack of efficacy and/or side effects. A very severe migraine-related disability was revealed using the MIDAS questionnaire (score of 140) and the HIT-6 test (score of 70). Therefore, according to the current national guidelines, treatment with erenumab up to 140 mg was started, and at the three-month follow-up, she noted a significant reduction in migraine days (8 days/months) and migraine-related disability (MIDAS score of 30 and HIT-6 score of 48). Nevertheless, given the history of drug-resistant migraine, the a brain MRI was performed that showed multiple punctate WMHs in the deep white matter and confluent lesions in the periventricular white matter associated with enlarged perivascular spaces (Figure 3). On the basis of the neuroimaging hallmarks, the patient was admitted to our neurological leukoencephalopathy clinic. The general and neurological examinations were unremarkable. A complete inflammatory/autoimmune/infective work-up on blood and cerebrospinal fluid was negative. No coagulopathy was detected either. Similarly, no pathological findings were found on trans-thoracic echocardiography, carotid duplex ultrasound, visual evoked potentials, or cervical spine MR. Genetic screening for familiar headache and cerebral small vessel diseases was then conducted, revealing a pathogenic heterozygous *NOTCH3* gene mutation: *c.665G>A (pCys222Tyr)* on exon 19, which confirmed a diagnosis of CADASIL. Treatment with erenumab was continued due to the substantial clinical benefit without adverse events, and after one year of treatment, the patient reported to have migraine only 5 days per month (thus reverted to episodic migraine) and a significant reduction in migraine disability burden, with a MIDAS total score of 8 and a HIT-6 test score of 40. The brain MRI was repeated, and no new lesions were found.

## 4. Discussion

Migraine is highly frequent in CADASIL and up to one-third of CADASIL migraine patients has been reported to have a severe disability because of migraine refractoriness to standard-of-care medications [11]. The treatment of migraine in this rare disease is based on clinical experience and empirical data, as indicated in a recently published literature review and meta-analysis [10]. There are some concerns regarding the use of several drugs for CADASIL treatment, either for episodic or chronic migraine, due to their potential vasomodulatory influence in patients with precarious cerebrovascular autoregulation or to other adverse effects in this disease [10]. Triptans and ergot derivatives, for example, act with cerebral vasoconstriction and could cause injury to the capillary endothelium, with an unknown safety profile in this setting [2,18]. Beta-blockers, amitriptyline, and topiramate could worsen mood and cognitive disturbances [14], and, in particular, beta-blockers showed a high rate of unfavorable responses [10]. On the other hand, common analgesics, opioids, and calcium channel blockers did not show unfavorable responses during CADASIL migraine attacks [10], and valproate seemed to have a therapeutic potential in the acute setting [10,18]. Acetazolamide had a good effect in terms of migraine prophylaxis [18], and the detection of hyper-homocysteinemia, a novel proposed risk factor for stroke and cerebral small vessel disease, in CADASIL patients [2,18], suggests the possibility of using vitamin B supplementation as a new beneficial treatment [18]. In this scenario, available therapeutic strategies for CADASIL remain inadequate [18]. 

In this paper, we reported our experience with two CADASIL patients treated with a CGRP receptor antagonist. CADASIL was genetically diagnosed in both patients after the drug’s introduction based mainly on MRI findings and after excluding other causes of leukoencephalopathy. Both cases presented a disabling drug-resistant migraine; indeed, several preventive treatments had been used without efficacy. When erenumab was introduced, for both patients, there was a drastic reduction in the frequency and duration of attacks (days with headache changed from 17 to 8 in patient 1 and from 18 to 5 in patient 2 after 12 months), and the intensity of pain, with a significant improvement in the quality of life (MIDAS and HIT-6 scores reduced from 116 and 63 to 6 and 59 in patient 1 and from 140 and 70 to 8 and 40 in patient 2 after 12 months). Neither patient presented adverse events and brain MRI repeated at a 1-year follow-up did not show new lesions. 

In a comprehensive search conducted on the Embase and MEDLINE databases, we found only one previously reported CADASIL patient treated with erenumab [19]. For this study, we used the following keywords: “erenumab”, “galcanezumab”, “fremanezumab”, “eptinezumab”, “anti-CGRP,” and combinations with “CADASIL”, “Cerebral autosomal dominant arteriopathy with subcortical infarcts and leukoencephalopathy”, “CADASIL migraine”, and “CADASIL headache”. Analogously to our cases, a previously reported 58-year-old CADASIL patient suffering from migraine with aura started erenumab, after the failure of several preventative treatments, with a significant reduction in headache severity and aura length and without evidence of ischemic complications [19].

CGRP and its receptor are involved in nociception [20] and are highly prevalent in the vasculature, preserving cardiovascular homeostasis in pathophysiological conditions [17]. Hence, CGRP may serve as a vasodilatory protective mechanism during episodes of cerebral and cardiac ischemia. Anti-CGRP drugs act mainly on the trigeminal ganglion and in other region lacking a blood–brain barrier [11], although their effectiveness on potentially central symptoms of migraine has recently been reported [21,22,23]. Some concerns have been raised regarding the potentially harmful effect of blocking CGRP-mediated vasodilation; in principle, CGRP blockage could increase stroke risk even in the general population. For patients at a higher risk of stroke, including those with a monogenetic predisposition like CADASIL, this aspect is particularly important to consider. Indeed, reduced cerebral blood flow with an impaired cerebral hemodynamic reserve has been reported in the disease as well as impaired endothelium-independent VSMC relaxation, including a lower increase in dermal blood flow after capsaicin application, a stimulus that induces a neurogenic inflammatory response caused by the predominant release of calcitonin gene-related peptide (CGRP) [20,24]. Blocking the CGRP system could potentially and particularly raise the vascular risk in this group of patients. Recently, an expert viewpoint claimed that in the absence of more extensive data, this therapy should be avoided in CADASIL patients [20].

However, clinical trials and observational studies conducted on migraine patients taking anti-CGRP drugs did not show a significant change in stroke risk in the general population [25]. Moreover, in a cohort of 60 migraine patients who were on erenumab, cerebral vasomotor reactivity and brachial flow-mediated dilation were specifically studied by means of a transcranial Doppler (evaluating the change in the mean flow velocity after breath-holding test) and a Doppler ultrasound (measuring the change in brachial artery diameter after reactive hyperemia), respectively [26]. These two techniques measure different aspects of arterial regulation and are not related to each other. Vasomotor reactivity reflects the dilatation of intracranial arterioles in response to vasodilatory stimuli, such as hypercapnia, while flow-mediated dilation reflects the arterial capability to self-regulate its tone through an endothelial response to changes in the local environment. In this study, erenumab treatment for 4 months did not exert any effect on either the cerebral vasomotor reactivity or peripheral flow-mediated dilation, supporting the hypothesis that the drug does not interfere with vascular tone [26]. 

In our patients and in the one previously reported, erenumab did not result in any cardio- or cerebrovascular event during a relatively long period of treatment. There was a considerable reduction in monthly migraine days, acute medication use, and disability, with a considerable improvement in quality of life reported. However, anecdotal evidence should be carefully evaluated for its inherent limitations.

## 5. Conclusions

To best of our knowledge, we have reported on the second and third CADASIL patients treated with erenumab for drug-resistant migraine, showing a reduction in the frequency and severity of attacks, with a consequent improvement in quality of life and without any sign of worsening ischemic cerebral disease or other adverse event. Given the rarity of CADASIL, recruiting a sufficient number of patients for clinical trials on the effectiveness and potential complications of CGRP/R antibodies is highly unlikely. In the meantime, this treatment should be carefully considered as a therapeutical strategy for CADASIL-related migraine, preferably only in drug-refractory migraine with severe disability, clearly discussing with the patient, and under strict clinical and neuroimaging surveillance. 

## Data Availability

Data supporting the findings in the present study are reported in the article. The raw data collected and analyzed are available from the Corresponding Author on reasonable request.
